# A somatic reference standard for cancer genome sequencing

**DOI:** 10.1038/srep24607

**Published:** 2016-04-20

**Authors:** David W. Craig, Sara Nasser, Richard Corbett, Simon K. Chan, Lisa Murray, Christophe Legendre, Waibhav Tembe, Jonathan Adkins, Nancy Kim, Shukmei Wong, Angela Baker, Daniel Enriquez, Stephanie Pond, Erin Pleasance, Andrew J. Mungall, Richard A. Moore, Timothy McDaniel, Yussanne Ma, Steven J. M. Jones, Marco A. Marra, John D. Carpten, Winnie S. Liang

**Affiliations:** 1 Translational Genomics Research Institute, Phoenix, Arizona, USA; 2 Canada’s Michael Smith Genome Sciences Centre, British Columbia Cancer Agency (BCCA), Vancouver, BC, V5Z 4S6, Canada; 3 Illumina Cambridge, Ltd., Saffron Walden, UK; 4 Illumina, Inc., San Diego, California, USA

## Abstract

Large-scale multiplexed identification of somatic alterations in cancer has become feasible with next generation sequencing (NGS). However, calibration of NGS somatic analysis tools has been hampered by a lack of tumor/normal reference standards. We thus performed paired PCR-free whole genome sequencing of a matched metastatic melanoma cell line (COLO829) and normal across three lineages and across separate institutions, with independent library preparations, sequencing, and analysis. We generated mean mapped coverages of 99X for COLO829 and 103X for the paired normal across three institutions. Results were combined with previously generated data allowing for comparison to a fourth lineage on earlier NGS technology. Aggregate variant detection led to the identification of consensus variants, including key events that represent hallmark mutation types including amplified *BRAF* V600E, a *CDK2NA* small deletion, a 12 kb *PTEN* deletion, and a dinucleotide *TERT* promoter substitution. Overall, common events include >35,000 point mutations, 446 small insertion/deletions, and >6,000 genes affected by copy number changes. We present this reference to the community as an initial standard for enabling quantitative evaluation of somatic mutation pipelines across institutions.

Dramatic developments in genomic technologies in the past decade have seeded the flourishing of next generation sequencing (NGS) applications in both the research and clinical laboratory settings. While the feasibility of identifying mutations using whole genome, whole exome, and targeted DNA sequencing has been demonstrated, a gold standard somatic reference set remains undefined. Such a reference is needed to enable interpretation of results generated using analytical pipelines that may differ significantly across institutions and to account for bias or variability in sample preparation and sequencing.

In order to define references to support the implementation of sequencing in the clinic, the National Institute of Standards and Technology (NIST) has established the Genome in a Bottle (GIAB) Consortium. By integrating fourteen sequencing data sets generated from the NA12878 cell line using five different technologies and that were analyzed using multiple aligners and variant detection tools, they defined a benchmark set of genotypes^1^. Additionally, Illumina’s Platinum Genome project has publically released sequencing data and analysis of a three-generation seventeen-member CEPH (Centre d’Etude du Polymorphisme Humain; Utah residents with northern and western European ancestry) pedigree (1463) in order to evaluate the accuracy of variant calling^2^. However, a similarly well-characterized somatic reference set for whole genome sequencing data has yet to be established. Previous studies have contributed to this undertaking by performing analytical and clinical validation of DNA sequencing^3^,^4^,^5^,^6^, comparing the performance of mutation callers^7^,^8^,^9^, and publically releasing somatic alterations identified from paired tumor/constitutional cell lines available from ATCC (www.atcc.org)^10^. In the latter study, Pleasance *et al*. performed whole genome sequencing (WGS) on COLO829, an immortal cell line derived from a metastasis from a cutaneous melanoma patient, and COLO829BL, a lymphoblastoid line from the same subject. By reporting on an extensive catalogue of somatic alterations in COLO829, this study set an early foundation for outlining a somatic reference.

The hypermutated nature of the COLO829 genome and accessibility of the COLO829 tumor and normal cell lines through ATCC, along with this pre-existing dataset make COLO829 a good candidate cell line on which to base a somatic reference set. We thus performed additional cross-institutional sequencing of paired COLO829/COLO829BL PCR-free NGS libraries on the Illumina HiSeq platform. With the intention of defining a somatic standard, this approach takes into account the expected variability across different cell passages, library preparation approaches, sequencing platforms, and informatics pipelines. We present here the first report of a multi-institutionally defined somatic reference standard using the paired COLO829/COLO829BL cell lines which incorporates previously published results from the original COLO829 somatic analysis^10^.

## Results

### Creation of a somatic reference standard dataset

**Figure 1:**
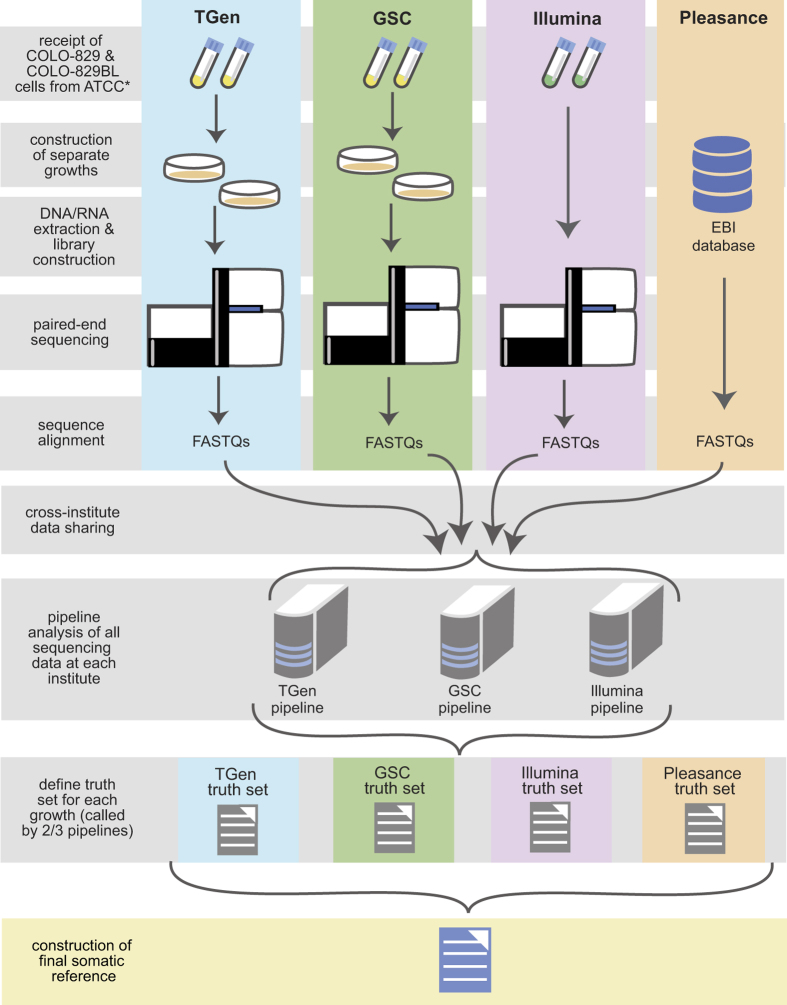
Overview of data generation and collection. *Illumina acquired extracted DNA from ATCC for library construction, sequencing, and analysis.

**Figure 2:**
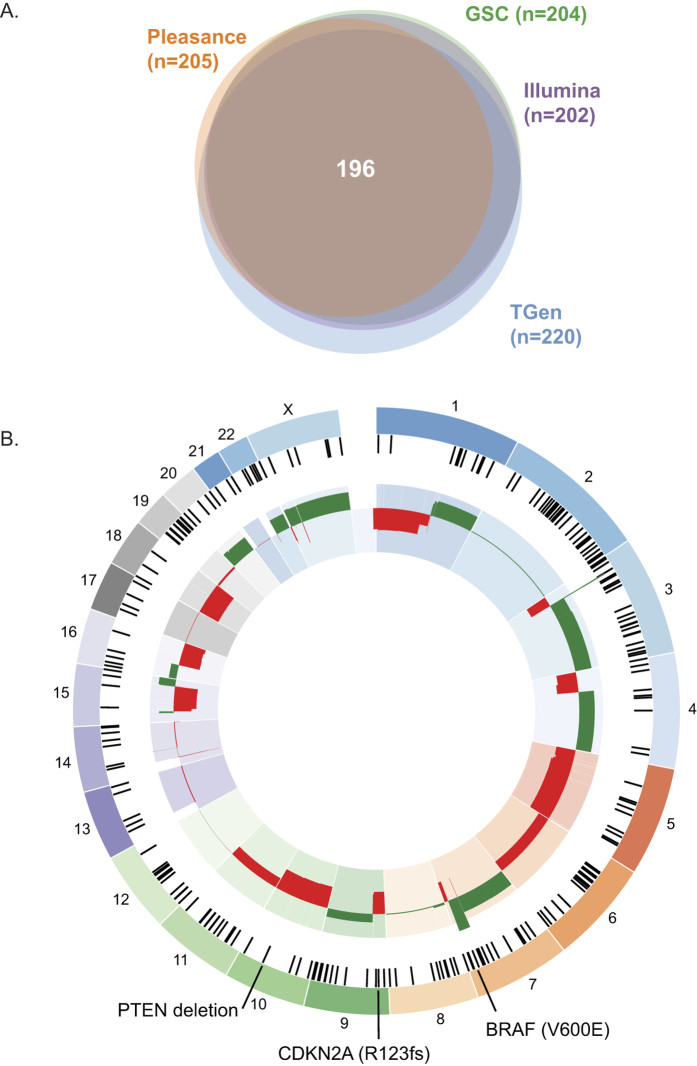
Construction of a somatic truth set for COLO829. (**A**) Identification of somatic reference SNVs. The total numbers of coding SNVs present in each truth set are shown. (**B**) Final somatic reference standard. Selected events are shown. Somatic coding SNVs are shown as black tick marks within the outermost ring. Consensus CNV gains are shown in green and consensus CNV losses are shown in red in the innermost circle.

WGS metrics and summary findings for each institution are listed in Table 1. Over 18 billion mapped reads were generated across all PCR-free whole genome data sets, including data generated from Pleasance *et al*.^10^, from the Translational Genomics Research Institute (TGen), Canada’s Michael Smith Genome Sciences Centre (GSC) in British Columbia, and from Illumina, Inc. Mean mapped sequence coverages of properly paired reads of 83X was obtained for both COLO829BL and COLO829 across all four data sets with the 2010 Pleasance data set demonstrating the lowest coverages. An overview of data generation and collation is shown in Fig. 1. To construct the somatic reference standard, data generated from DNA from separate cell culture preparations of the tumor/normal pair were independently analyzed by the TGen, GSC, and Illumina analytical pipelines. Consensus variants were compiled as a gVCF (genome variant call format) to distinguish true positives, true negatives, and no calls within each lineage. Somatic variants called by at least 2 out of the 3 pipelines were first selected to generate the true positive truth set for each of the four cell preparations. Somatic events commonly called across all four truth sets were used to generate the progenitor somatic reference standard (Fig. 2B). Under circumstances whereby a somatic event is present in at least 3 of 4 truth sets and for which the fourth truth set has low coverage (depth of coverage [DOC]<20 reads), the alteration was also included in the somatic reference standard.

Overall, a union of 49,337 somatic single nucleotide variants (SNVs) was identified across all truth sets with 35,543 SNVs falling within the final reference set following filtering as described above (Methods; Fig. 1). Breakdowns of all identified somatic SNVs and indels (insertion/deletions) across institutions are summarized in Table 2. Identified SNVs and indels encompass multiple classes including synonymous, missense, and UTR (untranslated region) events. Of note, generation of the reference standard is impacted by the amount of data generated by each institution. Analysis of discordance, as defined by the failure to detect a variant due to lower DOC, is shown in Supplementary Figure 1. For somatic small indels, a union of 735 events was identified with 446 indels falling within the final reference after filtering (Methods; Fig. 1). Pleasance *et al*. originally reported 32,325 total point mutations (292 coding somatic SNVs) and 983 small indels (no coding somatic indels)^10^, thereby demonstrating the divergence of separate cell growths and emphasizing the need to incorporate data from separate passages to define true positive somatic variants. The ratio of non-synonymous (missense or loss of function mutations) to synonymous SNVs for coding point mutations in the somatic reference standard was 1.69 (whereas Pleasance *et al*. reported 1.78) indicating limited selection on non-synonymous base substitutions.

Several notable somatic coding alterations are present within the reference (Supplementary Table 1). This includes the common *BRAF* (B-raf proto-oncogene, serine/threonine kinase) V600E (Val600Glu) mutation, which was previously also reported^10^. Missense mutations impacting the kinase domain of *BRAF*, including the common codon V600 mutation, occur in 30–72% of malignant melanomas^11^,^12^,^13^. Importantly, the reference created here also contains a 2-base pair deletion within the *CDKN2A* (cyclin-dependent kinase inhibitor 2A) coding sequence (R123fs), which was not initially detected in the Pleasance data set. This event was subsequently reported to be present in the Pleasance data set only following a targeted evaluation of *CDKN2A*^10^. Among other observations was an *FZD7* (frizzled class receptor 7) P285S mutation that was not originally reported^10^, but which was manually confirmed in the Pleasance data, albeit at a low DOC. This particular mutation was supported by 8 reads in the Pleasance data, in comparison to 94, 79 and 52 reads in the Illumina, TGen, and GSC truth sets, respectively (Supplementary Figure 2).

The somatic reference presented here also includes 150 somatic SNVs falling within 3′ UTRs and 26 within 5′ UTRs, as annotated by our group. We also identified a dinucleotide base substitution (chr5:1, 295, 228: CC > TT) in the *TERT* (telomerase reverse transcriptase) promoter across all pipelines and data sets except that of Pleasance *et al*., due to low coverage (sequence depth at chr5:1, 295, 228: Illumina = 45, Pleasance = 5, TGen = 49, GSC = 19). Mutations in the *TERT* promoter have been described in 71% of melanomas^14^,^15^, and also have been found to occur in additional malignancies including hepatocellular^15^,^16^ and central nervous system tumors^16^,^17^,^18^,^19^. In the final somatic reference, we additionally observed a promoter mutation in *NDUFB9* (NADH dehydrogenase (ubiquinone) 1 beta subcomplex, 9, 22 kDa; chr8: 125, 551, 344: C > T), which was previously described in COLO829^20^ and is present in 4.4% of melanomas^21^. This particular somatic base substitution interrupts a transcription factor binding motif 20 and may thus impact *cis*-regulatory mechanisms.

Considerable aneuploidy was observed across the genome. Both chromosome and arm level gains and losses were observed for all chromosomes except chromosome 2 and 12 (Supplementary Figure 3; Supplementary Table 2), with copy number alterations impacting over 6,500 genes. Individual institutional CNV plots are shown in Supplementary Figure 3A and a summary of all CNVs are shown in Supplementary Figure 3B. In addition, focal events were observed across all lineages with 9 genes impacted by focal deletions and 181 genes impacted by focal amplifications. Genes commonly impacted by focal gains or losses include *BRAF* which is amplified by two copies over a 24.6 megabase region on chr7q31.33–36.1. A 12 kb focal deletion was also observed within *PTEN*, presumably leading to loss of function. The CNV gain impacting *BRAF* was previously reported in COLO829^10^ and the region of *PTEN* loss overlaps with a region of homozygous loss in the original report^10^. While the *PTEN* loss is also reported in COSMIC for COLO829, no copy number changes were identified to impact the *BRAF,* or *CDKN2A*, loci for the COLO829 tumor in COSMIC^22^. Moreover, Pleasance *et al*. reported copy gains encompassing the *PDGFRA* (platelet-derived growth factor receptor, alpha polypeptide), *KIT* (v-kit Hardy-Zuckerman 4 feline sarcoma viral oncogene homolog), and *KDR* (kinase insert domain receptor) loci on the short arm of chromosome 4, which similarly showed gains across all analyzed cell line lineages in this study.

### Meta-analysis of the somatic reference standard

**Figure 3:**
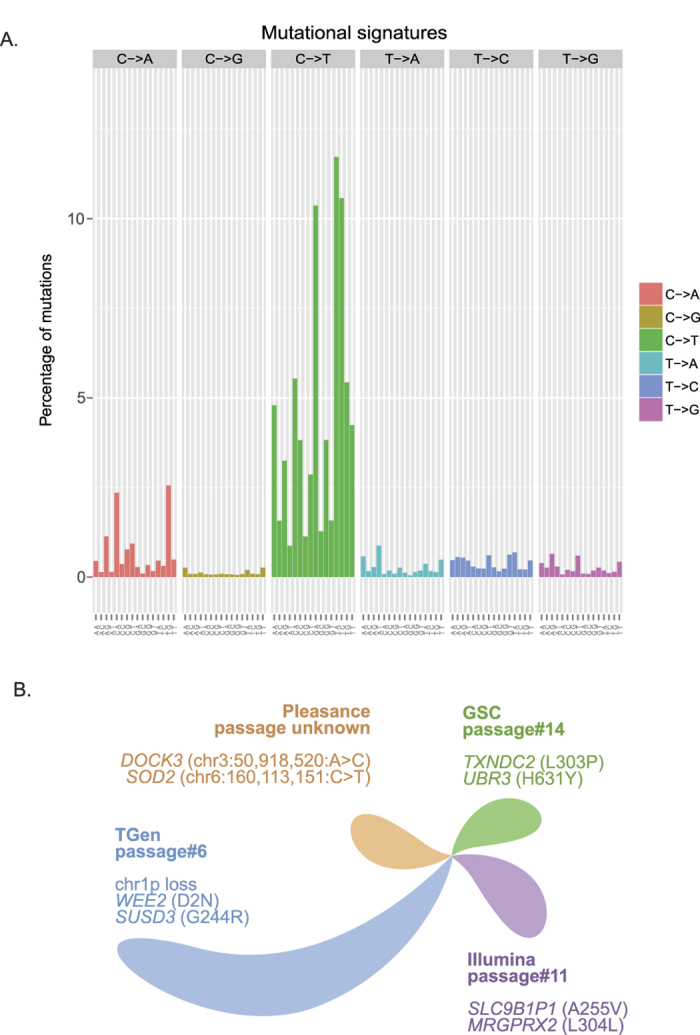
Meta-analysis of the COLO829 somatic reference. (**A**) Mutational signature of all SNVs. All substitutions are referred to by the pyrimidine context of the mutated base pair. (**B**) Genomic evolution of COLO829. A schematic of unique somatic alterations across each truth set is shown. Separate lobes reflect the level of divergence demonstrated by each truth set as measured by the number of unique somatic SNVs and indels for that data set. Examples of unique events are shown. The Pleasance sample did not demonstrate unique coding SNVs or indels (chromosomal coordinates of unique intronic events are shown).

Evaluation of the somatic reference standard indicated that the most common somatic base substitution is C > T transitions (72.8%; Fig. 3A). 50.6% of all substitutions were comprised of C > T transitions occurring in a dipyrimidine context. This percentage falls short of the 60% threshold that characterizes the presence of the ultraviolet (UV) light signature reported in malignant cutaneous melanomas^23^,^24^,^25^,^26^, and that which was described originally in COLO829^10^, to indicate that changes to the UV signature accumulated in the separate lineages. The highest incidence of C > T transitions occurred in the T*A trinucleotide context (16.1% of all C > T transitions), followed by the T*C context (14.5% of all C > T transitions). The second most frequent base substitution was C > A transversions (10.9%), which is previously described to be associated with DNA damage caused by reactive oxygen species^10^,^27^. Overall, the somatic reference has an average of 12 mutations per megabase, which falls within the prevalence range previously reported for melanoma^28^. CNVs (Fig. 2B; Supplementary Figure 3) were also widespread and thus indicate chromosomal instability in COLO829, as expected due to the highly mutated state of the line.

Overall, variants in the final reference standard parallel the *BRAF* subtype of cutaneous melanomas, the largest genomic sub-group reported by TCGA^21^, and which is defined by point mutations at *BRAF* V600 and K601. The reference presented here additionally has wild-type *TP53* (tumor protein p53), a feature of approximately 90% of *BRAF* subtype melanomas^21^. The patient from whom the COLO cell lines were derived from was forty-five years of age, which is in line with the observation that patients with *BRAF* subtype melanoma are younger (primarily ranging from 15 to 60 years of age) compared to other molecular subtypes including RAS, NF1, and triple wild-type subtypes of cutaneous melanoma^21^.

### Genomic evolution of COLO829

Meta-analysis of all data sets revealed the presence of somatic events unique to separate cell line lineages that were sequenced at each institution, reflecting genomic changes occurring through cell divisions. Cell line passages of sequenced samples varied across institutions (TGen: passage 6 for COLO829 and passage 8 for COLO829BL, GSC: 14 for COLO829, 3 for COLO829BL; Illumina: 11 for COLO289, passage was not provided by ATCC for COLO829BL; Pleasance: not available). Analysis of the union of all SNVs and indels across all data sets revealed that the most similar samples are the Illumina and GSC samples, and the TGen and Pleasance samples. The TGen sample demonstrated the greatest divergence compared to the remaining data sets with 3296 unique somatic SNVs and indels (Fig. 3B), of which 34 (1.0%) consisted of coding alterations (non-synonymous, synonymous, splicing altered). Coding events include *WEE2* (WEE1 homolog 2; D2N), *SUSD3* (sushi domain containing 3; G244R), and *KCNC1* (potassium channel, voltage gated Shaw related subfamily C, member 1; G404E), Divergence of the TGen sample was also evidenced by a unique homozygous deletion (Supplementary Figure 3: chr1: 554,900–571,700) that was not present in the other three samples. The Pleasance sample demonstrated 716 unique somatic SNVs and indels, none of which were coding alterations. The GSC sample demonstrated 727 unique mutations, which includes 6 (0.8%) coding events (missense, splicing altered, synonymous). These events include *TXNDC2* (thioredoxin domain containing 2; L303P), *UBR3* (ubiquitin protein ligase E3 component n-recognin 3 (putative); H631Y), and *MOSPD1* (motile sperm domain containing 1; P16S). Lastly, the Illumina sample demonstrated 1067 total unique SNVs or indels, including 3 (0.3%) coding alterations (*MRGPRX2* (MAS-related GPR, member X2; L304L), *EGFLAM* (EGF-like, fibronectin type III and laminin G domains; N220D), and *AC187652.1/FAM20C* (family with sequence similarity 20, member C; Q7L)).

## Discussion

In this study, we define a reference standard for cancer sequencing by performing multi-institutional sequencing analysis of the paired melanoma/normal COLO829/COLO829BL cell lines. At TGen, GSC, and Illumina, separate samples were prepared using PCR-free DNA library preparations, sequenced on the Illumina HiSeq, and analyzed using analytical pipelines developed separately at each institution. This approach mitigates expected bias that may be introduced at different steps in the workflow, and also minimizes variability that may be introduced during PCR enrichment through the generation of PCR-free whole genome libraries. The reference we present here, which also incorporates data from Pleasance *et al*.^10^, is comprised of 35,543 SNVs, 446 indels, and over 6,500 genes impacted by concurrent copy number changes. Several genes were associated with multiple events, such as *BRAF* V600E which was focally amplified and *CDKN2A* which contained both a small frameshift indel and was part of a larger copy number loss. Notably, because the original Pleasance data set had lower average mapped coverages compared to the other three data sets, true positive events in the final standard also include base substitution events that are called across three of four truth sets under circumstances whereby a mutation may not be detected in the fourth truth set due to lower DOC. Conversely, true negatives in the final standard are defined as alterations that are not supported by a minimum DOC of 20. Particularly relevant for future applications, this tumor/normal cell lines contains hallmark clinically relevant mutations spanning different mutation classes, including a *BRAF* V600E SNV, a *PTEN* 12 kb focal deletion, a *TERT* dinucleotide block substitution, and a 2 bp small deletion in *CDK2NA*.

As expected, we identified variability between the final reference standard presented here and the original Pleasance study. Divergence was demonstrated by the absence of somatic mutations in the original study, including the *TERT* promoter dinucleotide base substitution and the *CDKN2A* deletion, both of which were called across the remaining three truth sets from TGen, GSC, and Illumina. Such variability is derived from a spectrum of factors including lower coverages obtained in the original study, differences in pipelines used, the use of PCR-free approaches to generate libraries in this study, possible differences in experimental procedures, genomic variation introduced from cell line divisions, and variable error profiles resulting from the use of distinct sequencing technologies^29^ as the original study performed sequencing on the Illumina GAIIx platform. Somatic base substitutions and small indels unique to each truth set were also identified, as reflected through the analysis of the union of all somatic SNVs and indels detected across all four truth sets. Meta-analysis of the union of SNVs indicated that the GSC and Illumina data demonstrated the highest correlation whereas the TGen data set, with the lowest known passage number, was the most divergent. Theoretically, it is expected that cell lines that have undergone more passages would accumulate a greater number of mutations. However, our results suggest that variable conditions surrounding passaging of cells may have introduced a larger abundance of alterations in the TGen cell line. Although it is unclear as to precisely what factors may have caused such divergence, this feature is important to capture during construction of a gold standard given the demonstrated variability in the genomic background of different passages of the same cell line. These findings highlight that genomic alterations may be initiated through cell divisions within the same cell line and thus emphasize the need to perform validations on later passages.

As previously mentioned, variability in each truth set may result from a number of potential factors. While the same library preparation kit and sequencing platform were used across institutes, with the exception of that from Pleasance *et al*., biases, in addition to bias associated with sequencing different cell passages, may have been introduced at each site. Potential origins of variability may be attributed to differences in sample quantitation, any variation in temperature during end repair or ligation, variation in enzyme performance across different lots of kits, etc. Additionally, variable input amounts of DNA were used to construct libraries across TGen, GSC, Illumina, and Pleasance. It is, however, relevant to capture such factors into construction of the reference standard so that the final standard may be extrapolated for use at other institutions and laboratories. Furthermore, internal pipelines used at TGen, GSC, and Illumina also demonstrated discrete differences in sequence data analysis and variant calling. Both TGen and GSC use BWA-MEM^30^ for sequence alignment, whereas Illumina utilized iSAAC^31^. In addition, both GSC and Illumina utilize Strelka^32^ for SNV and small indel detection, whereas TGen pinpoints SNVs by identifying variants called by two out of three callers (MuTect^33^, Strelka^32^, Seurat^34^) and identifies small indels from an intersection of Strelka and Seurat calls. Lastly, because all data was generated on the Illumina HiSeq platform, sequencing errors that are characteristic of Illumina’s technology may also introduce biases in the reference. Potential sources of errors that may impact base-calling include overlapping of emission signals (crosstalk), formation of mixed clusters, phasing and pre-phasing issues, and signal decay which causes an increase in error rates towards the end of reads^35^. However, in this study, integration of data generated from separate institutions, as well as sequencing to higher depths, aids to mitigate base-calling errors that may have arisen in the separate data sets generated at each institution. Overall, the construction of the somatic reference standard presented here normalizes against many differences by defining true positive somatic genomic events in COLO829. The availability of these lines will enable further refinement of this initial reference set of variants through sequencing of COLO829 on additional platforms.

With the continued implementation of genomic technologies in both research and clinical laboratories, the utility of establishing molecular standards and references is clear. Adoption of a somatic reference standard to support the identification of tumor-specific alterations in whole genome sequencing data will allow for consistent reporting of somatic events and more widespread adoption of WGS. This will subsequently allow for more consistent interpretation of results both to better understand the genomic background of tumors and also to identify potential therapeutic targets. With a reference that is constructed and vetted by multiple institutions and pipelines, biases can be minimized and results generated from different laboratories can thus be compared. A few caveats are that the COLO829 cell lines may continue to evolve such that regular molecular checks will be required. However, with the reference presented here, the generation of a set of targeted panel controls, that can be analyzed in parallel with paired tumor/normal samples, is a possibility and would thus represent a more cost efficient alternative for adopting whole genome somatic controls. Such controls can be prepared and sequenced alongside research and clinical samples, and analyzed in parallel to support data evaluation and interpretation. Another caveat is that prior to adoption of a somatic reference by laboratories, individual testing will initially be required to ensure the performance of the standard. Although further vetting will be needed, the somatic reference standard we present here represents the first step towards defining a common control for somatic analyses and thus sets the foundation for uniform molecular interrogation of cancer genomes.

## Methods

### TGen

#### Cell culture

COLO829 and COLO829BL were obtained from American Type Culture Collection (ATCC), Manassas, VA. Cells were maintained in RPMI 1640 and DMEM media, Life Technologies, Grand Island, NY, respectively. Media was supplemented with 10% heat-inactivated fetal bovine serum (FBS) and Antibiotic-Antimycotic, Life Technologies. Cells were incubated at 37 °C in a humidified 5% CO_2_ atmosphere and grown to ~80% confluency in T175 tissue culture flasks. COLO829 cells were harvested using trypsin and centrifugation. The non-adherent COLO829BL cells were pelleted by centrifugation. Cell pellets were resuspended in 5 mL of media and aliquots were counted using the Countess Cell Counter, Life Technologies, with the addition of trypan blue to measure viability. Aliquots were made in 15 mL conical tubes ranging from 5 × 10^6^ to 1 × 10^7^ cells per tube and cells were pelleted by centrifugation and immediately flash frozen and stored at −80 C until ready for DNA isolation.

#### DNA isolation

The Qiagen AllPrep DNA Mini Kit, Qiagen, Valencia, CA, was used to isolate nucleic acids from the COLO829 and COLO829BL cell pellets. Specifically, 600 μl of Buffer RLT plus was added to the thawed pellets to disrupt the cells. The lysates were transferred to QiaShredder columns, Qiagen, for homogenization. Genomic DNA purification was conducted as directed by the AllPrep DNA/RNA Mini Handbook, Qiagen. DNA was quantified using the Qubit 2.0 Fluorometer (Life Technologies) and the Nanodrop spectrophotometer (Thermo Scientific; Waltham, MA), and 260/280 and 260/230 absorbance ratios were evaluated for purity. DNA was also electrophoretically separated on a 1% TAE gel to verify the presence of high molecular weight DNA.

#### Library preparation and sequencing

1.1 μg of DNA from each line was used to generate whole genome libraries using the Illumina TruSeq DNA PCR-free LT Sample Prep Kit (Set A; cat#FC-121-3001) following the manufacturer’s protocol. Following sonication on the Covaris E210, 100 ng of each sample was electrophoretically separated on a 1% TAE gel to verify fragmentation. The remaining fragmented 1 μg of each sample was used to generate libraries. Final libraries were evaluated on the Agilent Bioanalyzer and quantitated by Qubit. Libraries were clustered onto HiSeq V3 rapid flowcells using the Illumina TruSeq Rapid PE Cluster Kit (2500; cat#PE-402-4001) and sequenced by synthesis on the HiSeq2500 (rapid mode) for paired 112 bp read lengths using Illumina TruSeq Rapid SBS Kits (2500; cat#FC-402-4002).

#### Sequencing data analysis

Pipeline analysis is triggered when data is written from the sequencer to the analysis server in the form of BCL files. Using a queuing system and write FAIL/COMPLETED system BCL files are converted to FASTQ files (raw sequence) and aligned to the genome using BWA-MEM^30^. Bwa-mem aligns long query sequences against a large reference genome utilizing a backward-search with a Burrows-Wheeler Transform tool. We used the reference genome from 1000 Genomes project^36^ build hs37d5 with decoy contigs [b37d5] and Ensembl v74^37^ for annotations.

After alignment, PCR duplicates are examined to locate duplicate molecules and are removed. Following local realignment, variant callers can be used to identify indels. Indel realignment is conducted using GATK 1.6^38^. Our framework uses tumor and constitutional samples to identify somatic variants. Variants include SNVs, small insertions, and small deletions present in the tumor but not in the germline DNA (COLO829BL). Mutect^33^, Strelka^32^, and Seurat 2.6^34^ were used to identify somatic mutations. SNVs called from at least two of the three callers are compiled to identify mutations. As Mutect does not call indels, indels called from both Strelka and Seurat were compiled to generate a final indel results list.

Copy number changes, or amplifications and deletions, are detected from coverage comparisons of tumor and germline data sets. Focal gains and losses were defined as occurring on segments that were less than 25 Mb in size and that have been amplified multiple times or deleted within the tumor sample. Chromosome level gains/losses were reported as gains and losses. Focal amplifications and deletions are detected with TGen’s internal copy number analysis (CNA) software^39^.

### GSC

#### Cell culture

Frozen vials of the metastatic melanoma cell-line COLO829 (CRL-1974) and EBV transformed B lymphoblast cells from the same individual (COLO829BL, CRL-1980) were purchased from ATCC via Cedarlane (Burlington, Canada). COLO829BL cells were cultured to passage #3 at 37 °C in RPMI-1640 media supplemented with Fetal Bovine Serum (10%). COLO829 cells were cultured to passage #14 at 37 °C in RPMI-1640 media supplemented with Fetal Bovine Serum (10%).

#### DNA extractions

Extraction of DNA from 16.7 million cells of COLO829BL and 17.4 million cells of COLO829 was performed using the Qiagen AllPrep DNA Mini Kit.

#### Library preparation and sequencing

To minimize genome library bias and coverage gaps associated with PCR amplification of high GC or AT-rich regions, we implemented an automated version of library construction using New England Biolabs’ Paired-end sample prep kit (E6000B) with Illumina TruSeq adapters. Briefly, one microgram of high molecular weight genomic DNA was arrayed in a 96-well microtitre plate and subjected to shearing to 300–600 bp by sonication for 30 seconds (Covaris). Sheared DNA was end-repaired and size selected using AMPure XP beads targeting a 300–400 bp fraction. After 3′ A-tailing, full length TruSeq adapters were ligated. Libraries were purified using AMPure XP beads and fragment sizes were assessed using an aliquot of PCR amplified library DNA on an Agilent 2100 Bioanalyzer DNA1000 chip, or Caliper GX DNA1000 chip. The PCR-free library concentration was quantified using a qPCR Library Quantification kit (KAPA, KK4824).

Tumor and normal genome libraries were sequenced with paired 125 bp reads using Illumina HiSeq2500 V4 chemistry running HCS2.2 controller software and RTA 1.18.61. The five lanes of each library generated 2.819 billion reads for the tumor and 2.968 billion reads for the normal.

#### Sequencing data analysis

Illumina paired end 125 bp whole genome reads were aligned to the reference genome GRCh37-lite (http://www.bcgsc.ca/downloads/genomes/9606/hg19/1000genomes/bwa_ind/genome) with the Burrows-Wheeler Aligner (BWA mem; version 0.7.6a)^30^. BAM files were sorted with SAMTools (version 0.1.13) and merged and duplicate marked using Picard MarkDuplicates.jar (version 1.71). Chastity failed reads were marked in the BAM files through a custom script using the Picard Tools API (version 1.31).

### Genomic SNV/CNV analysis

Somatic SNVs and small indels were identified in the genomic data with Strelka (version 1.0.6) and annotated against dbSNP 144, Ensembl 74, and COSMIC v76 using SnpEff (version 4.0f). CNV analysis was performed using a Hidden Markov Model approach to segment the genome into regions of consistent copy number^40^. The segments are based on tumor read counts in genomic bins to which an equal number of reads from the normal sample align.

### Illumina

#### Cell culture and DNA extractions

All cell culture and nucleic acid extractions were performed at ATCC following their standard protocol.

#### Library preparation and sequencing

500 ng of each DNA was prepared using the TruSeq DNA PCR-Free Sample Preparation Kit (Illumina^®^, cat# FC-121-3001) following the manufacturer’s protocol with modified Covaris DNA shearing settings: Duty cycle 10%, Cycles/Burst 200, Time 45 sec. The resulting libraries were sequenced on a HiSeq2500 in rapid mode with read length 101 × 101. Libraries were clustered on HiSeq flowcells and paired end sequenced on HiSeq2000/2500 instruments for paired 101 reads.

#### Sequencing data analysis

Data was aligned using iSAAC-01.14.02.06^31^ to a par-masked version of hg19 which does not include decoys from either bcls (Illumina data) or FASTQ files (Tgen, GSC and Pleasance). iSAAC is an ultrafast aligner which has been highly optimized to align next-generation sequencing data with low error rates. Germline Snps and small indels were called using iSAAC variant caller 2.0.17^31^ using a Bayesian framework to compute probabilities over diploid genotype states.

Small somatic variants were calling using Strelka 2.0.14^32^, which uses a Bayesian approach to represent continuous allele frequencies in both the tumor and normal samples whiles leveraging the expected genotype structure of the normal. Somatic structural variants were identified by Manta^41^ which combines paired-end and split read evidence to resolve variant breakpoints. CNV’s were called using SENECA v2.2.2.3^42^ which is a count based method which looks at the coverage differences between the tumor and normal. It calls CNVs with non-overlapping 1 kb windows which are then merged. The CNscore (p-value) is calculated using a transformed t-test by comparing the distribution of coverages around the CN breakpoint. The CN breakpoints are imprecise and the accuracy is estimated to be within 5 kb. A copy number of 2 represent no change, values above 2 represent a gain and values below are losses.

### Construction of the somatic reference standard

#### SNV and indel analysis

The truth set for each growth was constructed by compiling variants called from each pipeline. Small variants were normalized using vt-normalize^43^ to address the issues of left alignment and parsimony. We define an SNV variant biomarker as “chromosome:start location:reference:alternate”. Single Nucleotide Polymorphisms (SNPs) are combined based on their exact biomarkers. True positive indels consist of an exact biomarker match or an overlap match that may be displaced up to 10 base pairs to compensate for differences between callers. The final reference data set is created by calling all bases utilizing GATK’s Haplotype Caller^44^ in a genome wide mode. The final reference standard consists of variants that are present in all four truth sets or that are present in three of four truth sets when the fourth truth set demonstrates a DOC of < = 20 at the respective position.

#### Copy number analysis

We compiled segmented copy number changes from each pipeline and performed chromosomal segment to gene and state mapping. State is defined as a 3-tuple (loss, neutral, gain). For generality purposes we assume the cell line to be diploid, thus a copy number loss is a copy number lower than 2 copies, a gain is any increase over 2 copies, and neutral refers to 2 copies. CNVs called by at least 2 of 3 pipelines were selected as the truth for the individual growth and intersection across all growths were compiled for the final reference standard. Focal gains and losses were defined as occurring on segments that were less than 25 Mb in size and that have been amplified multiple times or deleted within the tumor sample.

#### Mutational signature analysis

Analysis of the final reference standard was performed using the Mutational Signature Analysis Tool (https://bitbucket.org/jtr4v/analysis-of-mutational-signatures)^28^. All substitutions are referred to by the pyrimidine context of the mutated Watson and Crick base pair. For each mutation, one upstream and downstream base is captured. This type of base capture leads to 96 possible mutations in the classification. All somatic reference SNVs except mutations with multiple alternate alleles were used to capture the mutational signature.

#### Data Access

All BAMs and VCFs, including that for the final somatic reference, can be accessed through NCBI’s (National Center for Biotechnology Information) dbGaP (database of Genotypes and Phenotypes; accession number phs000932) and the European Bioinformatics Institute (EGA (European Genome-phenome Archive) accession number EGAS00001001385).

## Additional Information

**How to cite this article**: Craig, D. W. *et al*. A somatic reference standard for cancer genome sequencing. *Sci. Rep.* **6**, 24607; doi: 10.1038/srep24607 (2016).

## Supplementary Material

Supplementary Information

Supplementary Table 1

Supplementary Table 2

## Figures and Tables

**Table 1:** WGS metrics.

		**TGen data**		**GSC data**		**Illumina data**		**Pleasance data**	
		**COLO829BL**	**COLO829**	**COLO829BL**	**COLO829**	**COLO829BL**	**COLO829**	**COLO829BL**	**COLO829**
	Mapped reads	2,226,992,988	2,214,792,580	2,626,814,448	2,516,031,030	2,569,616,093	2,890,861,222	1,703,131,862	2,092,759,741
	Mapped paired Reads	2,215,137,161	2,204,283,295	2,600,711,902	2,494,669,592	2,564,928,975	2,888,165,980	1,694,413,815	2,083,919,389
	Median insert size	341	341	400	409	348	336	197	203
	% Alignment	99.0	99.4	99.0	99.1	99.0	99.1	99.3	99.4
	Average coverage	79.42	79.37	101.51	97.22	129.19	120.41	24.29	37.56
	Properly paired reads	2,183,906,643	2,180,616,978	2,518,297,733	2,422,792,278	2,542,369,674	2,864,033,284	1,684,269,960	2,066,734,682
	Mapped bases	246,067,396,020	247,815,655,476	324,367,737,235	310,765,322,484	258,096,280,820	290,447,668,499	126,036,007,551	154,431,022,209
TGen Pipe	Ti/Tv Ratio	4.255		3.88		3.97		4.08	
	dbSNP rate	0.06		0.0435		0.0465		0.0663	
	#somatic SNVs called	42,122		39,805		40,438		34,695	
	#somatic indels called	3,505		6,939		6,315		36,635	
GSC Pipe	Ti/Tv Ratio	2.84		3.39		3.25		3.41	
	dbSNP rate	0.043		0.032		0.042		0.033	
	#somatic SNVs called	45,740		42,428		44,424		39,833	
	#somatic indels called	748		693		683		424	
Illumina Pipe	Ti/Tv Ratio	3.0058		3.51		3.49		3.59	
	dbSNP rate	0.046		0.039		0.042		0.037	
	#somatic SNVs called	421,901		40,683		41,287		34,050	
	#somatic indels called	597		547		546		327	

**Table 2:** Somatic alterations in the final reference standard.

SNVs	35,543
Stop gained	13
Splice acceptor	4
Splice donor	1
Missense	151
5′ UTR	26
3′ UTR	150
Synonymous	90
TF binding site	44
Intragenic	14,867
Intergenic	18,780
Upstream 5 kb of gene	770
Downstream 5 kb of gene	621
Splice region	26
Small Deletions	260
Frameshift	3
3′ UTR	3
Inframe deletion	2
Intragenic	109
Intergenic	126
Upstream 5 kb of gene	12
Downstream 5 kb of gene	5
Small Insertions	186
TF binding site	1
3′ UTR	2
Intragenic	84
Intergenic	91
Upstream 5 kb of gene	3
Downstream 5 kb of gene	5
Total	35,989
