# Supplementary material for: A somatic reference standard for cancer genome sequencing
Source: Sci Rep. 2016 Apr 20;6:24607. doi: 10.1038/srep24607 (PMC4837349; doi:10.1038/srep24607)

**A somatic reference standard for cancer genome sequencing**

David W. Craig^*^, Sara Nasser^*^, Richard Corbett, Simon K. Chan, Lisa Murray, Christophe Legendre, Waibhav Tembe, Jonathan Adkins, Nancy Kim, Shukmei Wong, Angela Baker, Daniel Enriquez, Stephanie Pond, Erin Pleasance, Andrew J. Mungall, Richard A. Moore, Timothy McDaniel, Yussanne Ma, Steven J. M. Jones, Marco A. Marra, John D. Carpten, Winnie S. Liang

***Supplementary Data***

**Supplementary Figure 1: Discordance in somatic SNV detection at variable read depth thresholds**


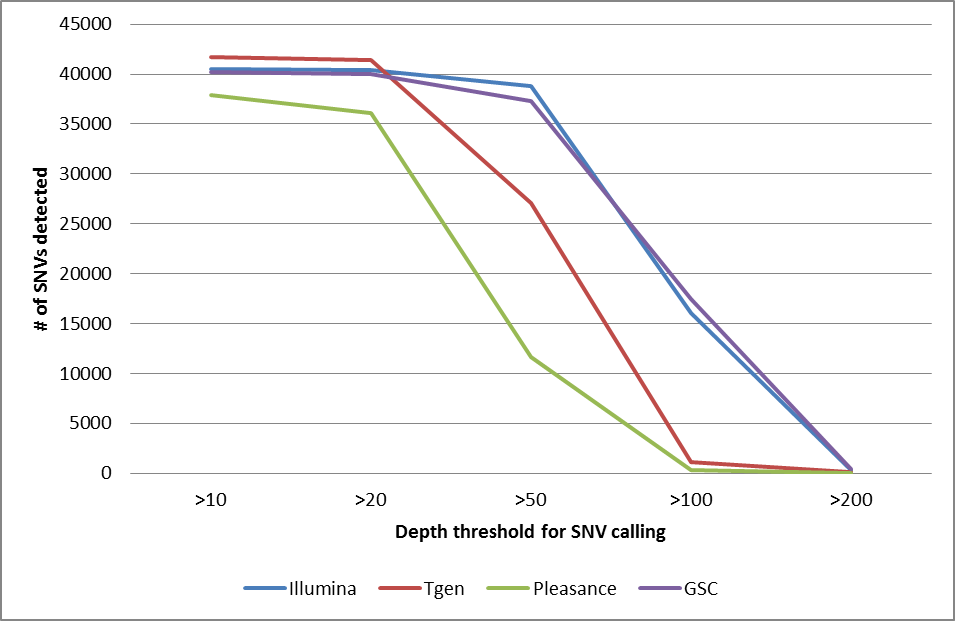


**Supplementary Figure 2: Evaluation of the somatic *FZD7* P285S mutation**

Tumor/normal BAMs for each truth set were visualized using the Integrated Genomics Viewer (IGV). The identified *FZD7* missense mutation is located at chr2: 202,900,223: C>T. All four tumor BAMs show reads supporting the presence of this mutation.

**Pleasance TGen GSC Illumina**


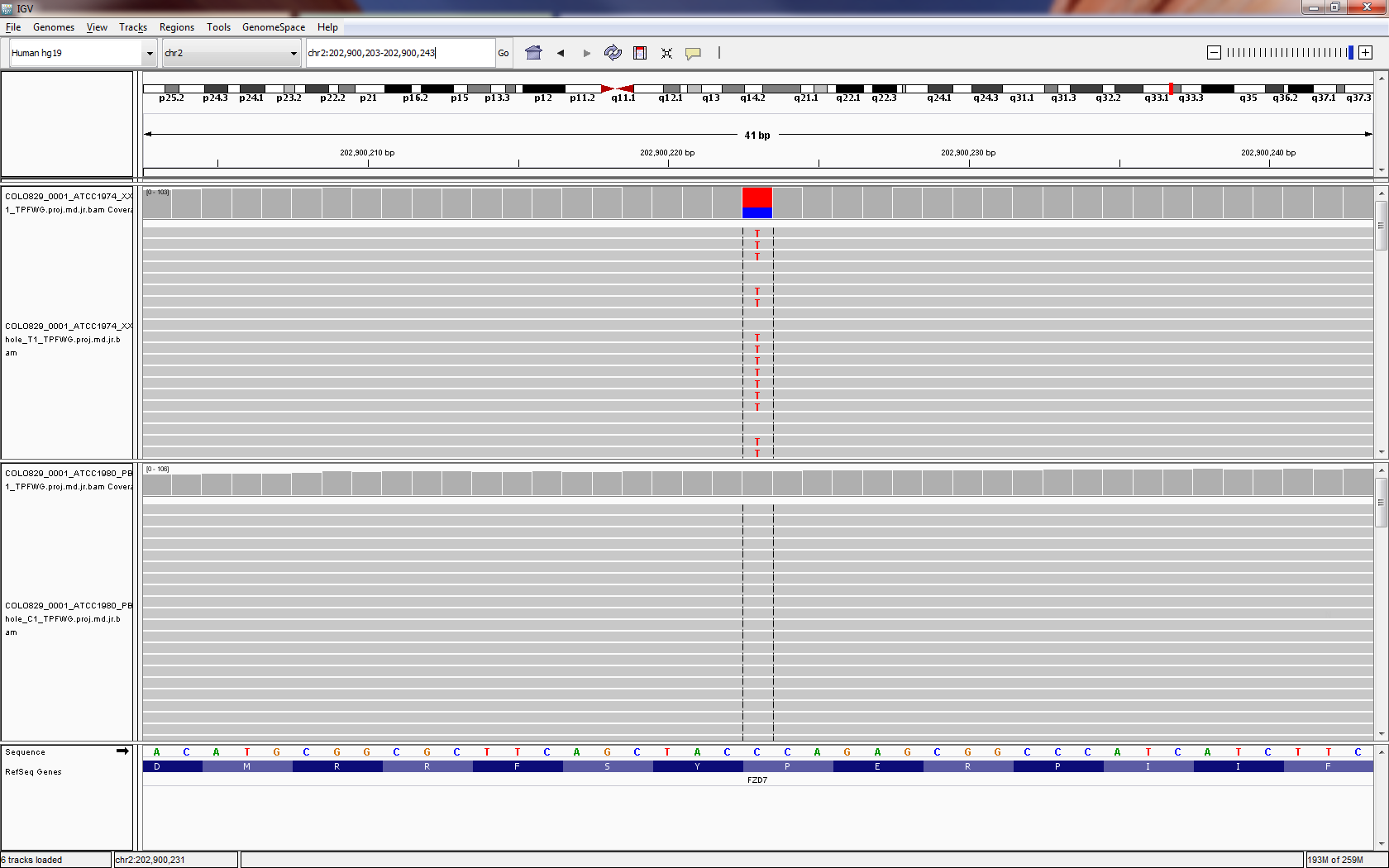

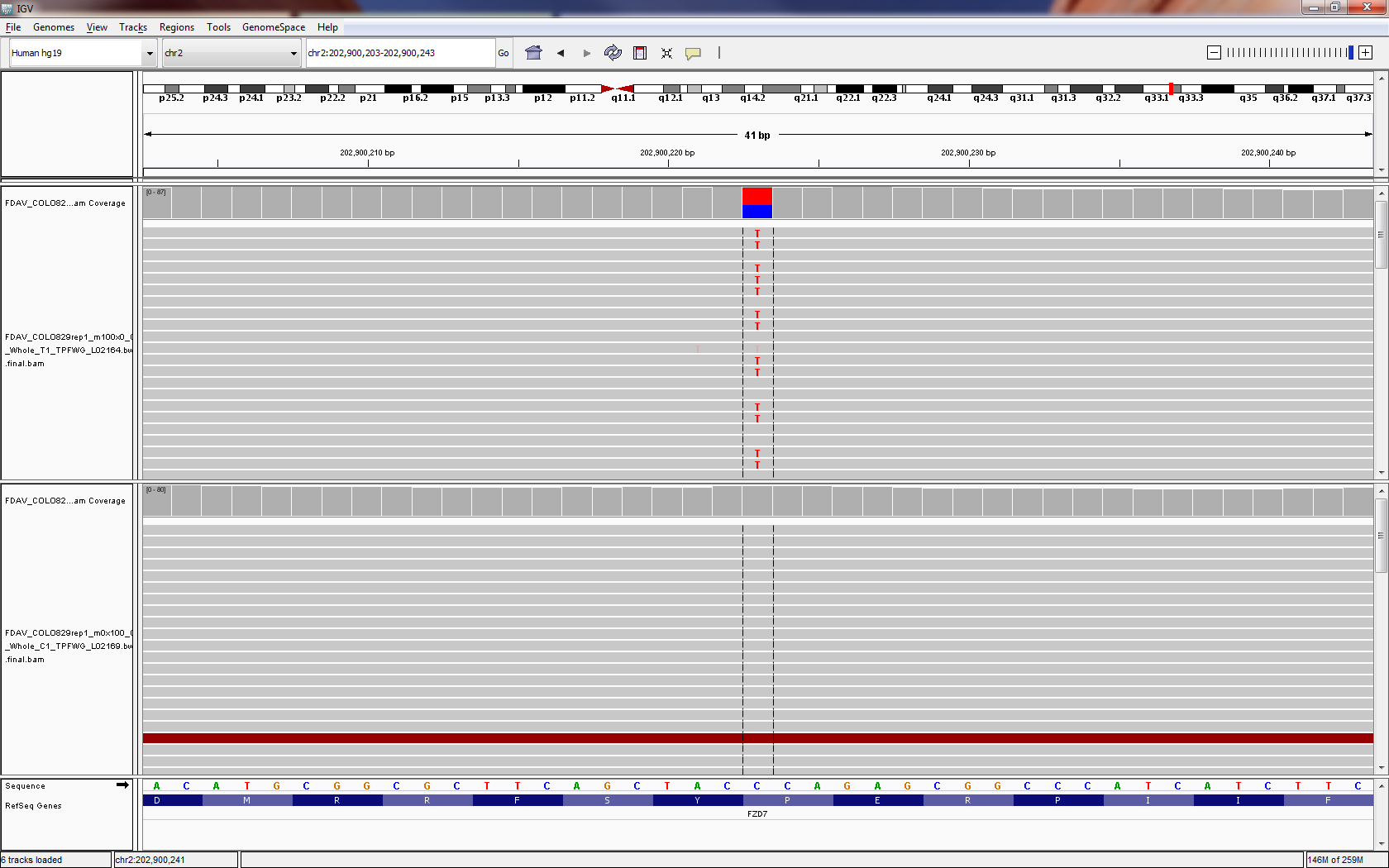

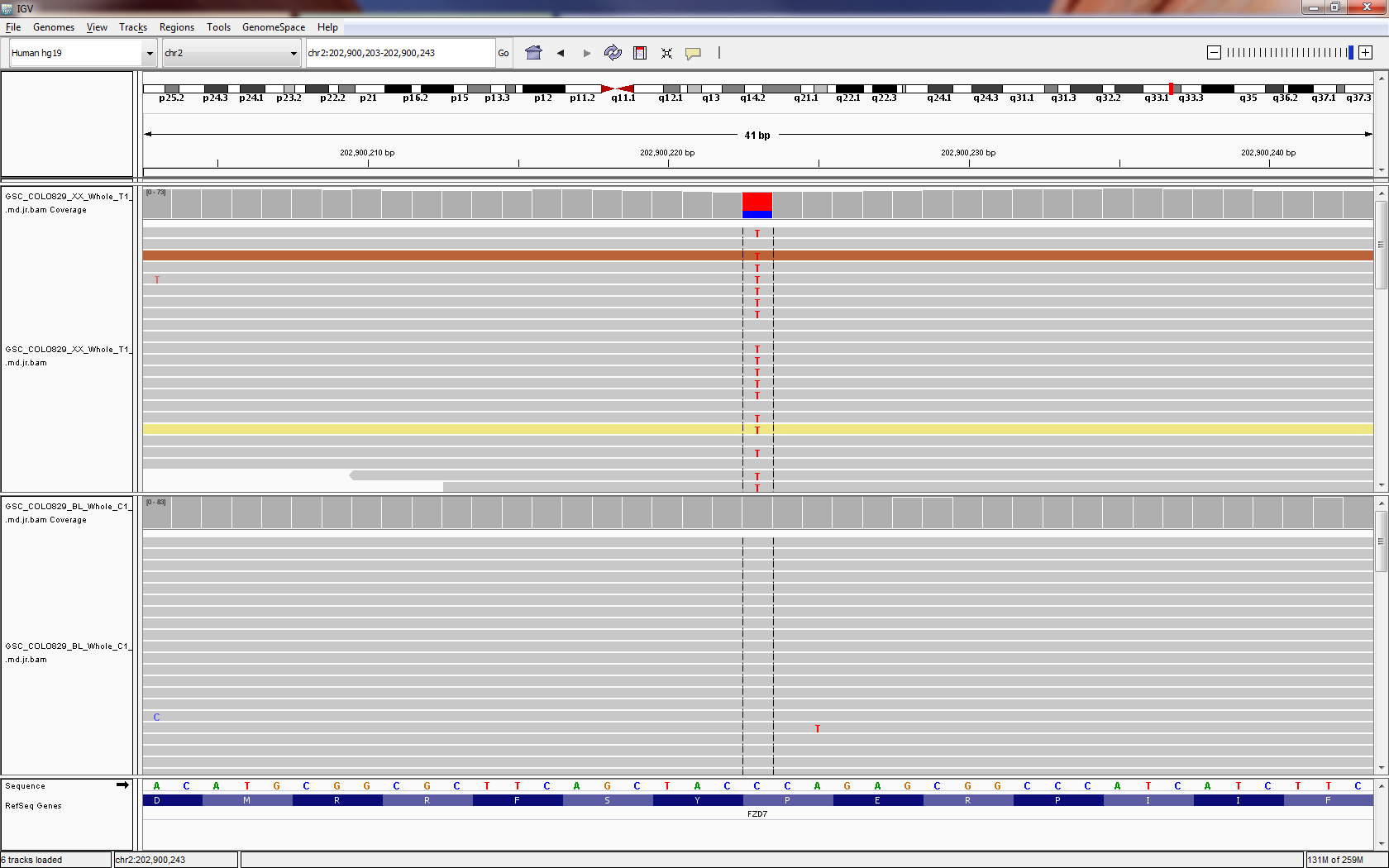

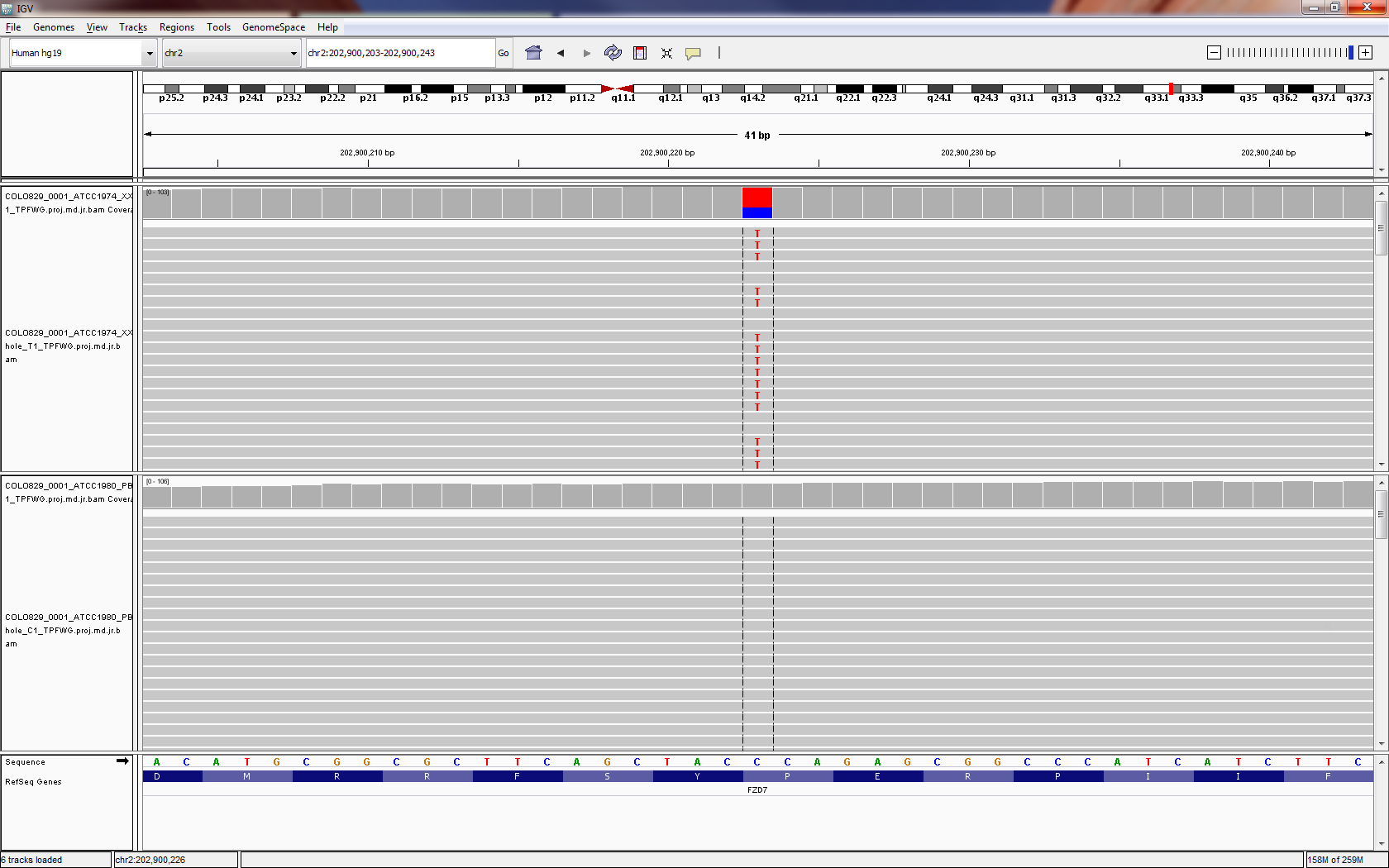


**Tumor**

**BAMs**

**Normal**

**BAMs**

**Supplementary Figure 3: CNV analysis**

**3A. Individual institutional CNV plots**

Individual CNV plots generated using the TGen analytical pipeline for each growth is shown.

(Green=CNV loss, red=CNV gain; X-axis=chromosome number, Y-axis=log fold change)


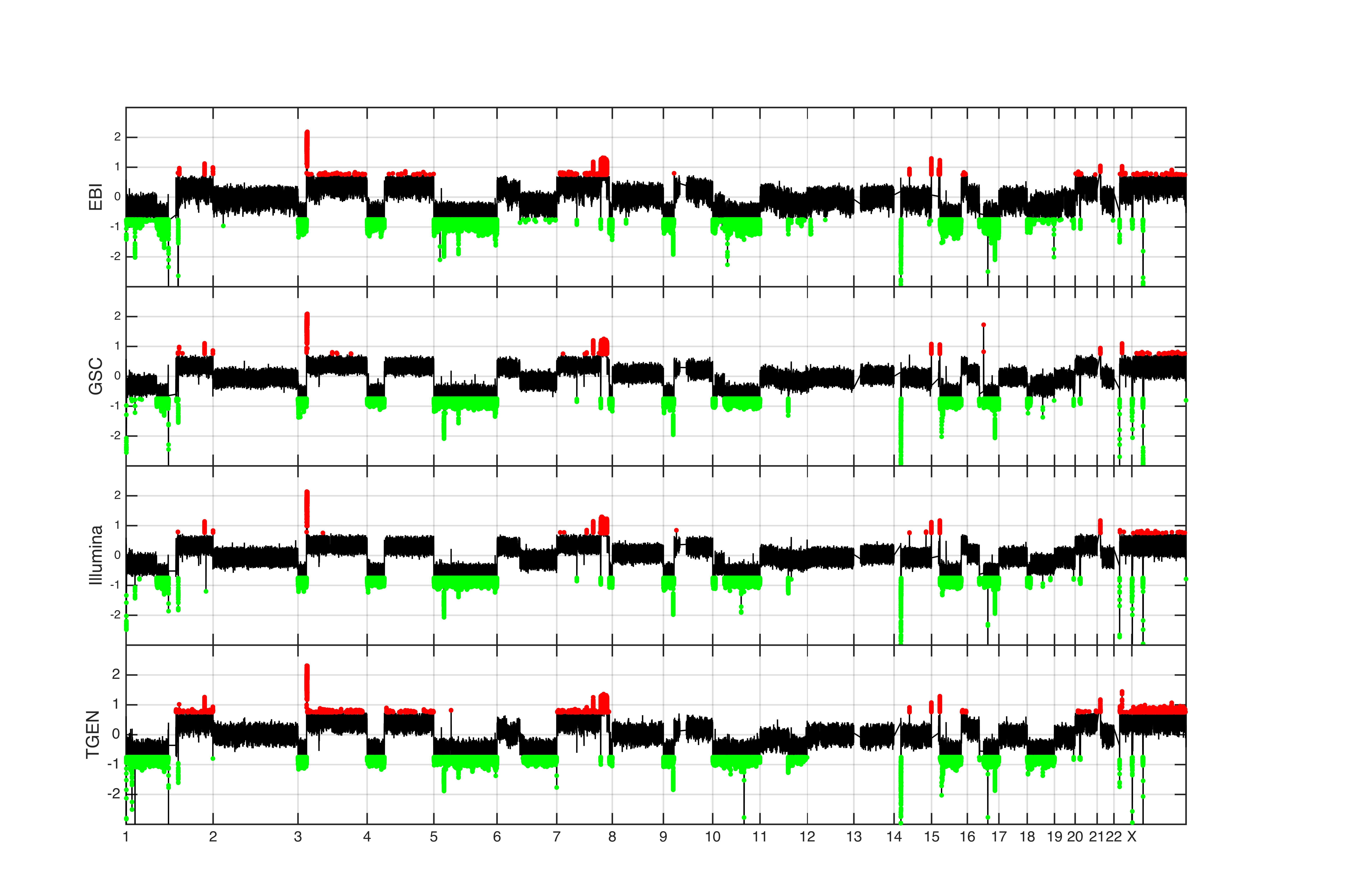


**3B. Summary CNVs**

All CNVs that were identified across all truth sets is shown (Y-axis=the number of truth sets with a copy gain (red) or copy loss (green) at that genomic location, X-axis=chromosomal location).


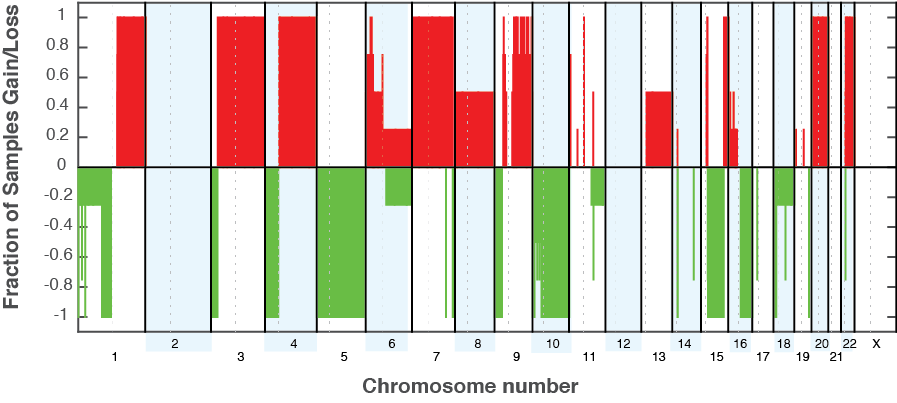

Supplement: Supplementary Information [file srep24607-s1.doc]
